# Probabilistic alignment leads to improved accuracy and read coverage for bisulfite sequencing data

**DOI:** 10.1186/1471-2105-14-337

**Published:** 2013-11-21

**Authors:** Changjin Hong, Nathan L Clement, Spencer Clement, Saher Sue Hammoud, Douglas T Carrell, Bradley R Cairns, Quinn Snell, Mark J Clement, William Evan Johnson

**Affiliations:** 1 Division of Computational Biomedicine, Boston University School of Medicine, Boston, MA, USA; 2 Department of Computer Science, University of Texas, Austin, TX, USA; 3 Department of Computer Science, Brigham Young University, Provo, UT, USA; 4 IVF and Andrology Laboratories, Departments of Surgery, Obstetrics and Gynecology, and Physiology, University of Utah School of Medicine, Salt Lake City, UT, USA; 5 Department of Oncological Sciences, Huntsman Cancer Institute, Salt Lake City, UT, USA

**Keywords:** DNA methylation, Bisulfite sequencing, Probabilistic alignment, Parallel processing

## Abstract

**Background:**

DNA methylation has been linked to many important biological phenomena. Researchers have recently begun to sequence bisulfite treated DNA to determine its pattern of methylation. However, sequencing reads from bisulfite-converted DNA can vary significantly from the reference genome because of incomplete bisulfite conversion, genome variation, sequencing errors, and poor quality bases. Therefore, it is often difficult to align reads to the correct locations in the reference genome. Furthermore, bisulfite sequencing experiments have the additional complexity of having to estimate the DNA methylation levels within the sample.

**Results:**

Here, we present a highly accurate probabilistic algorithm, which is an extension of the Genomic Next-generation Universal MAPper to accommodate bisulfite sequencing data (*GNUMAP-bs*), that addresses the computational problems associated with aligning bisulfite sequencing data to a reference genome. *GNUMAP-bs* integrates uncertainty from read and mapping qualities to help resolve the difference between poor quality bases and the ambiguity inherent in bisulfite conversion. We tested *GNUMAP-bs* and other commonly-used bisulfite alignment methods using both simulated and real bisulfite reads and found that *GNUMAP-bs* and other dynamic programming methods were more accurate than the more heuristic methods.

**Conclusions:**

The *GNUMAP-bs* aligner is a highly accurate alignment approach for processing the data from bisulfite sequencing experiments. The *GNUMAP-bs* algorithm is freely available for download at: http://dna.cs.byu.edu/gnumap. The software runs on multiple threads and multiple processors to increase the alignment speed.

## Background

DNA methylation is a chemical process in which a methyl group is added to the carbon-5 position of a DNA cytosine. In most vertebrates, DNA methylation typically occurs on the cytosine of a CpG dinucleotide [1,2], although some specific examples of other types of methylation have been shown to play roles in specific tissues [3-6]. Since its discovery over 60 years ago, DNA methylation has been linked to many important biological phenomena such as the suppression of gene expression [7,8], imprinting [9], X chromosome inactivation [10], epigenetic reprogramming during mammalian development [11], and cancer development [12]. Therefore, the study of genome-wide methylation patterns is currently of great interest to researchers, particularly in areas related to the molecular mechanisms of development, cancer, and chromatin dynamics.

When DNA is treated with sodium bisulfite, unmethylated cytosine residues are converted to uracil, while 5-methylcytosine residues are unaffected. Later in bisulfite sequencing (BS-seq) experimental protocols, PCR amplification or sequencing converts the uracil residues to thymines. The next step of finding the correct genomic location for a bisulfite-treated read (BSR) is a complicated and difficult process. Although cytosine to thymine changes are allowed for when mapping the BSRs to the genomic sequence, methylated and unmethylated CpG locations are often identified and, in some cases, it is impossible to distinguish between a bisulfite (BS)-treated thymine that originated from an unmethylated cytosine, and a true thymine from a different genomic location or an individual genomic variation at that location [13,14]. This ambiguity is often magnified by the presence of sequencing errors or low-quality bases. As a result, sophisticated computational strategies are required for aligning reads from BS-seq experiments.

The first whole-genome methylation profiles were performed on *Arabidopsis thaliana*. To map the resultant BSRs, alignment algorithms based on a probabilistic formulation and a suffix tree [15] as well as reference genome conversion [16] were used. However, it is not computationally feasible to apply this approach to larger genomes such as the human genome, or to experiments with the current deeper sequencing depths. Later algorithms, such as *BSMAP*[14], constructed seed tables of locations from both the original reference sequence and the BS variants, and then extends the seeds to form a possible mapping location. This seed extension process can be somewhat unreliable because the seeds must be exact and do not take into account BS-treated variations. BS alignment methods such as *BS Seeker*[17], *Bismark*[18], and *BRAT-BW*[19] have been used to map BSRs. These methods employ a Burrows-Wheeler transformation [20] for fast (in)exact string matching and then combine the results with either a pre-processing or post-processing script to handle BSRs with three letters after converting all Cs to T. The strength of these methods lie with reads with fewer mismatches, but they offer very limited support for aligning reads with insertions or deletions (indels). These methods also have difficulty in aligning Ts in the reads to Cs in the genome without also (incorrectly) aligning for cysteines in the reads to thymidines in the genome. More recently, the *LAST* alignment program has been adapted to align BSRs [21]. *LAST* uses a seed extension approach similar to the one used by NCBI BLAST [22], but the speed and mapping accuracy are increased by using variable-length seeds and base quality information [23].

In this study, we present a highly accurate probabilistic mapping algorithm, Genomic Next-generation Universal MAPper for Bisulfite Sequencing (*GNUMAP-bs*), for BSR alignment. *GNUMAP-bs* is an extension of the Genomic Next-Generation Universal MAPper (*GNUMAP*) [24] that can accommodate the alignment of BSRs to a reference genome. *GNUMAP-bs* was developed to achieve higher accuracy than other BS-seq approaches by including base quality scores in the alignment process. In addition, the *GNUMAP-bs* probabilistic mapping approach allows for the unbiased estimation of DNA methylation, especially when reads are aligned to multiple genomic locations.

## Results and discussion

We compared *GNUMAP-bs* with several commonly-used BS alignment methods, namely *BSMAP* (v2.3) [14], *Bismark*(v0.7.9) [18], *LAST*[21], and *BRAT-BW*[19]. We also compared *GNUMAP-bs* with the unpublished proprietary probabilistic aligner *Novoalign* (v2.07.09, http://www.novocraft.com) because it uses a probabilistic approach that is similar to the approach used in *GNUMAP-bs*. For a fair comparison, we considered only common options that were shared by all the alignment methods; otherwise, we used the default values or slight modifications of the defaults to achieve the best performance for each method. For each aligner, we allowed up to three or four base pair differences within 100-bp BSRs. For *GNUMAP-bs* and *Novoalign*, this resulted in cutoff values of -a 0.90 and -t 75, respectively. Considering that repeated genomic sequences often appear in mammalian genomes, we allowed up to 20 multiple mapping locations for each read. This approach to handling multiple alignments has only a small impact on the sensitivity of the more heuristic algorithms, but has moderate sensitivity gains in more traditional approaches like *GNUMAP-bs*. The *Bismark* software only reports up to two valid alignments. The parameter values that were used for each alignment method are given in Table 1.

**Table 1:** Parameters used in the two experiments for each of the aligners tested

**Mapper**	**Parameters**
*GNUMAP-bs*	-m 17 -s 1 -T 20 -a 0.90 (-a 0.92) -b
*Novoalign*	-k 17 -s 2 -r -A 20 -t 75 (-t 90) -b2
*BSMAP*	-n 0 -w 100 -v 3
*Bismark*	-n 2 -l 50
*Bismark-bt2*	-N 1 -L 20 –bowtie2 –min-score L,0,-0.6
*LAST*	-Q1 -j1 -d120 -n20 -f1 | last-map-probs.py -s150 -m0.95
*BRAT-BW*	default settings for single-end reads

Parameters in parentheses were used for the human BSR dataset. Up to 3 to 4 sequencing errors or mutations were allowed in a 100-bp long BSR. Because the human genome contains many repeated genomic regions, 20 valid mapping locations were allowed for a given read when the alignment method supported this feature.

We used two datasets to evaluate the performance of each of the alignment methods. The first was a simulated BSR dataset, which was carefully designed to mimic a typical human methylation experiment. The second was a real human BS-seq dataset, which was compared with an experimentally-derived human methylome profile.

### Simulated bisulfite sequencing experiment

For the simulation study, we randomly assigned 20% of the CGs in the whole human genome (NCBI build37/HG19) to represent unmethylated cytosines (Cs) by changing them to thymidines (Ts), thereby simulating complete BS conversion. For the remaining 80% of the CGs, 75% was randomly assigned to be fully methylated and, therefore the Cs remained as Cs. The remaining 5% was assigned to be methylated in proportions between 0.1 and 0.9. In this dataset, we assumed that all non-CG sites remained unmethylated, so these Cs were all changed to Ts for read generation. We used the *dwgsim* ( http://sourceforge.net/projects/dnaa/files/dwgsim) simulation tool with parameters -e 0.001-0.008 -1 100 -y 0.05 -r 0.002 -R 0.2 -C 10, to generate 100-bp BSRs with a 10? read depth across the genome. This simulation produced a BS-seq dataset that contained approximately 180 million (M) reads with a sequencing error rate that ranged from 0.001 to 0.008 and increased from the 5’ to 3’ ends, plus 5% randomly generated sequence, and a mutation rate of 0.002 (in which 0.2% were indels).

We found that the mapping sensitivities of the probabilistic aligners *GNUMAP-bs* (97.0%), *Novoalign* (96.3%), and *LAST* (96.9%), were higher than the sensitivities of *BSMAP* (93.9%) and *Bismark* (93.2%), as shown in Table 2. The *Bismark-Bowtie2* algorithm had the lowest false positive rate (0.1%), but at the cost of an approximately 7% decrease in its sensitivity. Overall, the *Bismark-Bowtie2* algorithm had 1.2 M fewer false positives than *GNUMAP-bs*, but approximately 10 M fewer BSRs were aligned correctly to the genome. The *Bismark-Bowtie* algorithm performed better than *Bismark-Bowtie2* in that it aligned 5 M more reads with only a slight increase in the number of incorrectly aligned reads. The *LAST* algorithm aligned nearly all the BSRs (99.2%) to the genome, but although the proportion of correctly mapped reads was similar to that of *GNUMAP-bs*, the error rate was much higher. *BRAT-BW* was not included in this comparison because the application software removed the original sequence header/name which contained information pointing to the correct alignment location of the BSRs.

**Table 2:** Simulated bisulfite read experiment

**Evaluation metric**	***GNUMAP-bs***	***Novoalign***	***BSMAP***	***Bismark***	***Bismark-bt2***	***LAST***
**Overall mapping results:**						
Total reads aligned (%)	156.6M(**97.8**)	155.8M(97.4)	153.4M(95.9)	149.5M(93.4)	145.4M(90.8)	158.7M(99.2)
Correctly aligned (%)	155.2M(**97.0**)	154.2M(96.3)	150.2M(93.9)	149.2M(93.2)	145.2M(90.7)	155.1M(96.9)
Incorrectly aligned (%)	1.4M(0.9)	1.7M(1.1)	1.5M(1.0)	0.3M(0.2)	0.2M(**0.1**)	3.6M(2.3)
**With ≥1 sequence variant:**						
Total reads aligned (%)	69.0M(97.8)	66.0M(93.6)	65.3M(92.6)	63.6M(90.2)	59.6M(84.4)	70.3M(**(99.7**)
Correctly aligned (%)	67.7M(**96.0**)	65.3M(92.6)	63.9M(90.1)	63.3M(89.8)	59.4M(84.1)	66.7M(94.6)
Incorrectly aligned (%)	1.3M(1.8)	0.7M(1.0)	1.4M(2.0)	0.3M(0.4)	0.2M(**0.3**)	3.5M(5.1)
**Predicted methylation:**						
Ave. absolute estimation err.	0.11	0.69	0.22	0.11	0.10	-
Standard err.	0.056	0.066	0.067	0.064	0.062	-
**Computational resource:**						
Total compute time (16 CPUs)	39 h 50 m	29 h 25 m	**4 h 28 m**	46 h 16 m	97 h 26 m	58 h 20 m
Peak memory usage (GB)	44.8	14.5	9.4	**5.9**	7.9	15.9
Reads per second per CPU	68	92	607	448	26	**753**

Simulation study of 160 million (M) simulated BSRs generated from the human genome reference sequence. The *GNUMAP-bs* algorithm was the most sensitive aligner, especially for reads with ≥1 sequence variant (sequencing errors or mutations). The *Bismark* algorithm had the smallest error rate with 1.2 M fewer erroneously assigned reads than *GNUMAP-bs*, however *GNUMAP-bs* correctly aligned 6 to 10 M more reads. The *BSMAP* algorithm had the fastest total run time, however its sensitivity was less than the sensitivity of the *GNUMAP-bs* algorithm. *LAST* mapped nearly all reads with a sensitivity that was comparable to that of *GNUMAP-bs*, but the mapping error rate for *LAST* was much higher than it was for *GNUMAP-bs*.

Differences in sensitivities of these methods were much more pronounced for BSRs that contained at least one sequencing error or genome variant, and the *GNUMAP-bs* sensitivity was clearly better than the sensitivities of the other approaches (Table 2). We also evaluated how accurately each aligner predicted the CG methylation levels when the true methylation level ranged from 10% to 90%. All the alignment methods tested performed well with mean absolute errors less than 0.01 and with standard deviations less than 0.07.

When the computational performances of each of the methods were compared (Table 2), we observed that *GNUMAP-bs* required the most RAM (44.8 GB) and *Bismark* required the least (5.9 GB). Because some of the software applications presented here support computation on multiple threads and some do not, we presented two different measures of computational speed: 1) the total run time on a 16 CPU linux server and 2) the number of reads processed per CPU per second. *GNUMAP-bs* required approximately 40 hours of total compute time to process the 180 M BSRs, while *BSMAP*, the fastest algorithm, was nearly nine times faster than *GNUMAP-bs*. The *LAST* application does not support parallel computing, so *LAST* had the longest total alignment time. However, the *LAST* algorithm aligned the most reads per second per CPU (753), while *Bismark-Bowtie2* aligned the least (26).

### Human BS-seq dataset

We also evaluated the performances of the alignment methods using a BS-seq dataset generated from samples from a healthy human donor collected at the Andrology Laboratory at the University of Utah (Salt Lake City, UT, USA). The BS-seq data were generated by coupling BS conversion and the Illumina HiSeq2000 platform, which generated 101-bp BSRs for analysis. We aligned 283.6 M reads from three lanes of BS sequencing data containing 85 M to 100 M sequencing reads each, to the recent build of the human genome (NCBI build37/HG19). The human BSRs were processed for quality control as suggested previously [25]. Briefly, the quality control involved masking low quality bases or trimming consecutive lowest quality bases at the 3’ ends of the reads.

We used the same parameters on these data as were used for the simulation experiment (Table 1) with two exceptions: -a 0.92 for *GNUMAP-bs* and -t 90 for *Novoalign*. The *LAST* algorithm again aligned the highest proportion (93.7%) of BSRs to the genome, followed by 70.0% for *GNUMAP-bs*, 68.2% for *Novoalign*, 67.1% for *BSMAP*, 67.0% for *Bismark*, 62.9% for *Bismark-Bowtie2*, and only 50.3% for *BRAT-BW*.

We compared these mapping results with the Sanger-based BS sequencing control data available from the Human Epigenome Project (HEP) [26] by selecting the data that were obtained using the same type of tissue as was used in our BS-seq dataset. The HEP data provides a natural gold standard for algorithmic evaluation. For this comparison, we focused on the 13,563 HEP CG sites on chromosome 22 (chr22), because these data provide the most comprehensive chromosomal CG coverage that is available in the HEP database. The data for the other chromosomes in the HEP showed similar profiles as chr22, but the coverage was much sparser.

Overall, the mapping results produced by the different alignment methods for the human BS-seq dataset were consistent with the results for the simulation dataset. Compared with *GNUMAP-bs*, *LAST* aligned almost twice as many reads to chr22 (3.02 M compare with 1.57 M); however, the CG read coverage for *LAST* (7.6 reads/CG) was only about 20% higher than for *GNUMAP-bs* (6.3 reads/CG) (Table 3). While *Novoalign* aligned more reads to and covered more CG sites on chr22 than *GNUMAP-bs*, *GNUMAP-bs* had better CG read coverage (6.3 reads/CG) than *Novoalign* (6.1 reads/CG) and the other aligners, *BSMAP* (5.8 reads/CG), *Bismark* (5.8 reads/CG), *Bismark-Bowtie2* (5.7 reads/CG), and *BRAT-BW* (5.0 reads/CG) (Table 3).

**Table 3:** Human bisulfite read experiment

**Evaluation metric**	***GNUMAP-bs***	***Novoalign***	***BSMAP***	***Bismark***	***Bismark-bt2***	***LAST***	***BRAT-BW***
**All chr22:**							
Reads Aligned	1.57M	1.65M	1.34M	1.31M	1.21M	3.02M	0.98M
CGs Covered	330K	336K	330K	310K	299K	373K	275K
CG read coverage	6.3	6.1	5.8	5.8	5.7	7.6	5.0
**HEP overlap:**							
CGs Covered	7,902	7,802	7,747	7,561	7,331	8,606	6,690
Correlation	0.887	0.889	0.887	0.888	0.895	0.882	0.894
Concordance	0.869	0.867	0.865	0.867	0.872	0.858	0.872
CG Read coverage	4.6	4.5	4.4	4.3	4.3	4.9	3.7

Study of 162.3 M Illumina BSRs on human chromosome 22 (chr22) from a human donor sample. The top rows show the overall mapping efficiency, including covered CGs sites and read coverage aligned to chr22. The bottom rows show the consistency of each mapping result with known methylation levels at 13,563 CGs sites on chr22 available from the HEP database. The concordance statistic is defined as the fraction of overlapped CGs covered by the aligner with HEP CGs for which the methylation prediction error was smaller than 0.25.

The differences between *GNUMAP-bs* and *LAST* become even less pronounced when the overlap between the mapped BSRs and the 13,563 CGs with HEP coverage on chr22 was considered. *Last* aligned the BSRs to 63.5% (8,606) of the CGs HEP sites compared with 58.3% (7,902) for *GNUMAP-bs*, and 57.5% (7,802) for *Novoalign*, 57.1% (7,747) for *BSMAP*, 55.7% (7,561) for *Bismark*, 54.1% (7,331) for *Bismark-Bowtie2*, and 49.3% (6,690) for *BRAT-BW* (Table 3).

**Figure 1:**
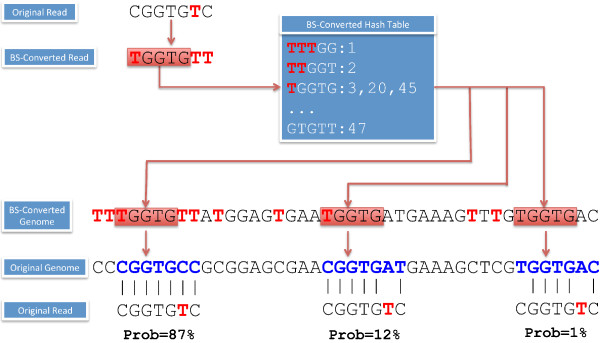
***GNUMAP-bs* workflow for mapping high-throughput bisulfite reads.** A flow-chart of the *GNUMAP-bs* algorithm is shown. For the reference genome, all Cs are converted to Ts and then each *k*-mer in the genome is hashed, producing a list of positions in the genome to which the *k*-mer sequence is mapped. Given a completely BS-converted read (e.g. TGGTGTT) where the corresponding original bisulfite read (BSR) is known (e.g. CGGTGTC), a query *k*-mer (e.g. TGGTG) is searched against the BS-converted hash table. Locations with a *k*-mer match to both the genome and the read are aligned using the original read and original genome and the *GNUMAP-bs* probabilistic Needleman–Wunsch (N-W) algorithm. If the alignment score passes a quality threshold, the location is considered a match and recorded on the genome for future output. The posterior probability is computed for each mapped location using Equation (1). Finally, all the mapped BSRs are used to quantify the fraction of methylation at each CG location across the reference genome.

To determine if increased coverage resulted in decreased alignment quality, we computed the correlation coefficient between the estimated HEP methylation levels and the methylation levels estimated from each alignment method. The correlation coefficients were 0.887 for *GNUMAP-bs* and ranged from 0.895 for *Bismark-Bowtie2* to 0.882 for *LAST* (Table 3). We also computed a concordance statistic for each of the methods as defined previously [27]. Briefly, the concordance is the fraction of sites for which the methylation levels (aligner vs. HEP) differ by less than a predefined cutoff (we used 0.25). Based on this statistic, we found that the performances of most of the aligners were similar, with concordance values of 0.869 for *GNUMAP-bs* and ranged from 0.872 for *Bismark-Bowtie2* 0.872 and *BRAT-BW* to 0.858 for *LAST* (Table 3). These results showed that the increased coverage levels produced by *LAST* result in lower consistency with the HEP data in terms of both correlation and concordance. Pairwise comparisons of the concordance for the CGs covered/not covered between *GNUMAP-bs* and the other aligners are shown in Figure 1. For example, Figure 1 shows 577 CGs that were covered by *GNUMAP-bs* but not covered by *Bismark*; the concordance of the *GNUMAP-bs* methylation estimates for these sites was 0.78. In contrast, only six CGs covered by *Bismark* were not covered by *GNUMAP-bs*, and the concordance for these sites was extremely low (0.33). These results clearly showed that the increased mapping coverage (for both numbers of CGs covered and reads per CG) obtained using *GNUMAP-bs* did not result in decreased alignment quality or methylation estimates.

## Conclusions

BS sequencing presents difficult challenges to researchers attempting to process the sequencing reads from BS-seq experiments. In this work, we present *GNUMAP-bs*, a highly accurate and effective alignment algorithm that is specifically designed to estimate DNA methylation levels with base-level resolution in BS-seq data. *GNUMAP-bs* uses a probabilistic approach to align BSRs to a reference genome. *GNUMAP-bs* was developed to achieve higher coverage and accuracy than other published BS-seq approaches by integrating base quality and alignment quality information in the mapping process. We have shown that the *GNUMAP-bs* probabilistic mapping approach results in an improved unbiased estimation of DNA methylation across the human genome. In simulated and real datasets, we showed that *GNUMAP-bs* outperforms other BS-seq alignment methods when both coverage and consistency were balanced with Sanger based BS sequencing controls.

In addition, *GNUMAP-bs* provides many high-demand features needed for constructing a high quality methylome from BS-seq data. First, *GNUMAP-bs* incorporates quality sequencing data into a dynamic programming framework. This feature gives *GNUMAP-bs* the best balance between sensitivity and specificity of the tested BSR aligners, especially for reads that contain short polymorphisms. Second, *GNUMAP-bs* adaptively assigns an optimal mapping stringency based on an effective read length after the original read is trimmed. Third, *GNUMAP-bs* not only relies on the maximal score alignment but also probabilistically considers suboptimal alignments; that is, the alignment score is converted to a posterior probability and the probabilistic scores quantify the likelihood of the true source location for each read across the reference genome. As a result, for both the simulated and real datasets, we showed that *GNUMAP-bs* was more effective that the other methods in detecting read locations in the presence of sequencing errors. *GNUMAP-bs* also displayed the highest consistency with a known HEP methylation database. Because *GNUMAP-bs* supports Message Passing Interface (MPI) processing, the computational burden of the dynamic programming can be alleviated. Moreover, with computer resources becoming cheaper, computing clusters with large numbers of nodes and cores, and more computing clouds are becoming available. Therefore, memory and CPU running times are less of a bottleneck, which is especially useful for *GNUMAP-bs* alignments. For this reason, accuracy should currently be a more important concern in BS-seq data analysis.

## Methods

**Figure 2:**
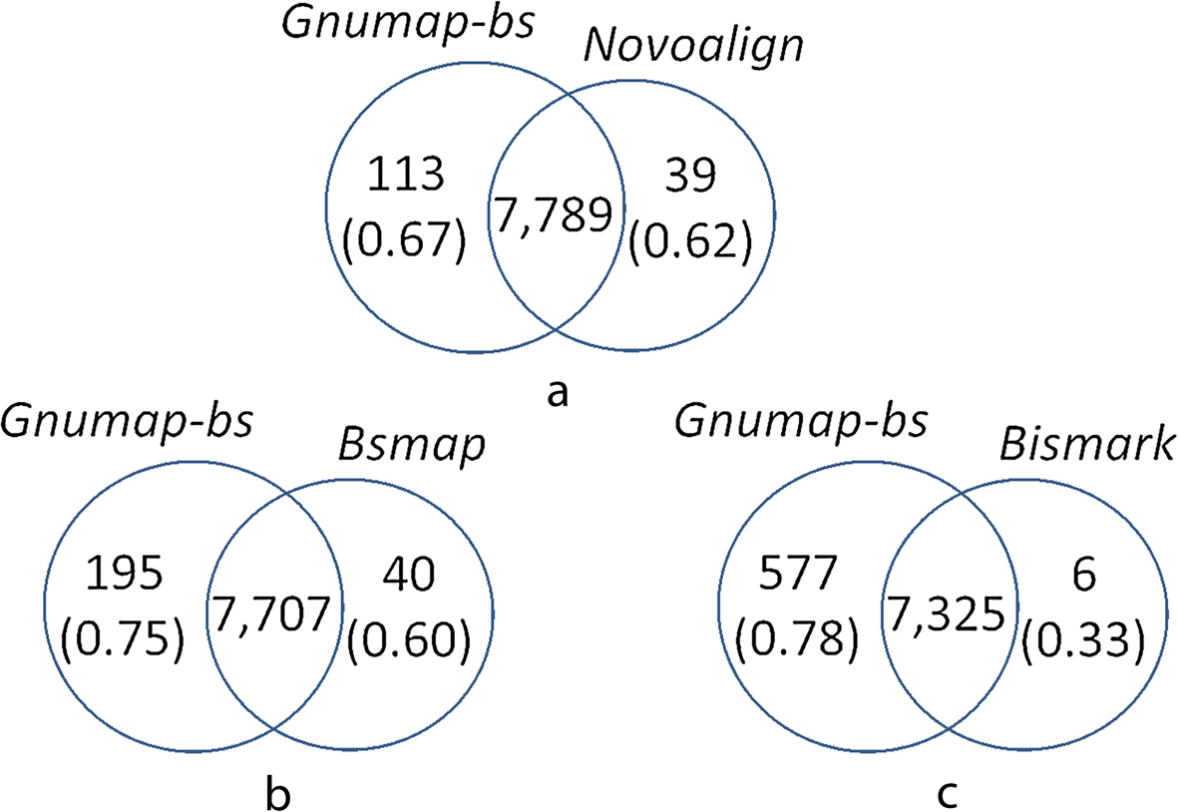
**Relative complement mapping consistency of *GNUMAP-bs * with HEP methylation profiles of human chromosome 22.** Venn diagrams between *GNUMAP-bs* and **(a)** *Novoalign*, **(b)** *BSMAP*, and **(c)** *Bismark* showing both the number of covered/uncovered CG sites and the concordance (in parenthesis) of these sites with the HEP methylation profiles. The estimated levels of methylation in the additional CG sites covered by the probabilistic aligners but not by the other aligners are highly concordant with the HEP results.

The *GNUMAP-bs* alignment algorithm is a modification of the *GNUMAP* algorithm, which consists of three main steps, all of which needed to be modified to align BSRs to a reference genome. A flow chart of the *GNUMAP-bs* algorithm is displayed in Figure 2. The first step is the construction of a hash table using all genomic subsequences, where the *k* nucleotide (nt) long (*k*-mers) are the keys and the hash table values store the genomic locations of the *k*-mer. In addition, *k*-mers from the BSRs are incrementally referenced in the reads in the genomic hash table. In *GNUMAP-bs*, the genome and the reads are artificially 'BS-converted’ by changing all Cs to Ts before the hashing step. This process ensures that all BSRs can be referenced into the hash table regardless of whether or not they contain methylated bases.

In the second step of the *GNUMAP* algorithm, all the BSRs are aligned to the genome at the hashed locations. The alignment is performed using a novel probabilistic alignment algorithm that uses base quality information. All the regions that meet a predefined alignment score threshold are retained for the final step. In *GNUMAP-bs*, the probabilistic alignment algorithm matches the unconverted (original) reads to the unconverted genome while allowing matches between Ts in the reads and Cs in the genome. Using dynamic programming in probability space for mapping BSRs to a genome has several benefits: 1) the Needleman–Wunsch algorithm is guaranteed to find the optimal alignment for a BSR; 2) by incorporating base qualities into the probabilistic algorithm, true DNA methylation can be more accurately identified in the alignment, especially in areas where the reads have low base quality; and 3) by making only a small change in the alignment scoring matrix, namely by removing the T (read) to C (genome) 'mismatch’ penalty and scoring these alignments as a 'match’, the probabilistic Needleman–Wunsch algorithm can accurately account for the BS changes in BSRs without losing reads that are partially converted, or that have genome variations or sequencing errors.

During PCR amplification after BS conversion, the C to T conversions in the BSRs lead to G to A conversions on the PCR-synthesized strand as described previously [14]. These G to A changes provide evidence of non-methylation on the original strand. The phenomenon also occurs for C to T changes on the PCR-synthesized strand. Therefore, the *GNUMAP-bs* algorithm maps each BSR twice, once looking for only C to T changes and then looking for only G to A changes. When a G to A change is observed, the read is considered to be a PCR-synthesized strand, and the non-methylation event is attributed to the original strand.

In the final step, the *GNUMAP-bs* algorithm quantifies the significance of an alignment (e.g. a posterior probability) by assigning a mapping score that is proportional to the relative quality of alignments and inversely proportional to the number of matching sites in the reference genome. Given a BSR, *r*, assume that there are *J* "best match" locations (potential mapping locations) for which the alignment score is *q*[ *d*] for alignment to genomic position *d*, which is also aligned to the first base pair location of *r*, matches some user-defined threshold. Then, the posterior probability at each location *P*(*r*_*d*_) can be computed as:

(1) $$P(r_{d})=\frac{e^{q[d]}}{\sum_{j=1}^{J}e^{q[j]}}$$

It is worth noting that the calibrated posterior probability is a special case of the *LAST*[21] mapping probabilities equation where a scaling factor for a bit score in the simple E-value statistics is not explicitly computed.

This probability provides an intuitive value between 0 and 1 representing the methylation signal for each read at each CG site. Other BS mapping programs use $\frac{1}{\#\text{mappings}}$ instead of Equation (1), which may not accurately capture the true posterior probability. The posterior probability indicates the relative significance of mapping a read to a particular position. The probabilistic Needleman–Wunsch computes a log-likelihood score; therefore, if a particular BSR has multiple possible mapping locations in the reference genome, the significance of each mapping decreases. After *GNUMAP-bs* computes *P*(*r*_*d*_) for each read, the algorithm combines the probabilities into one methylation profile vector and infers a true methylation ratio at each CG site.

For example, consider a nucleotide, *n*_*i*_ at genomic location *i*, and assume that *n*_*i*_ is a C residue in a reference genome. Let *C*^(*m*)^ be a methylated C residue and *L*(*r*) be the read length. For a set of aligned reads covering *i*, the methylation ratio *c*[*i*] can be computed as:

(2) $$c[i]=\frac{\sum P(r_{d}|n_{i}=C^{\left(m\right)})}{\sum P(r_{d})},d\leq i\leq d+L(r_{d}).$$

Intuitively, the reads that cover each C location across the reference genome provide evidence of true mapping so that a reliable methylation percentage can be obtained.

## Software implementation and availability

The *GNUMAP-bs* algorithm is integrated into the is *GNUMAP* software suite, and is freely available for download at http://dna.cs.byu.edu/gnumap. The *GNUMAP-bs* pipeline can accommodate single-end or pair-end reads in either FASTA or FASTQ file formats. A reference genome file (or multiple reference files) in FASTA format is also required. However, to increase the efficiency of the workflow, users can opt to write the reference genome hash table to a file, which can be used in future runs. *GNUMAP* outputs read alignments in standard SAM file format. The *GNUMAP-bs* software suite contains an addition application function (*sam2gmp*) that summarizes a SAM file and writes it into a text file that contains one line for each cytosine in the genome. Each line in this file contains the chromosome number, the location of the cytosine on the chromosome, the number of reads, and the numbers of As, Cs, Gs, Ts, and Ns covering the location. In addition, the text file gives a likelihood ratio p-value that indicates whether there is a significant number of Cs at the location (i.e. methylation significance).

In addition to the adaptations for BSRs, the *GNUMAP-bs* software also contains several modifications to reduce the computational time and memory needed for the alignments. For example, the initial read and genome conversion step reduces the genome alphabet to three bases, leading to an increase in the number of genome locations identified in the hash table. To reduce this effect, multiple seeds from each read are referenced by *GNUMAP-bs*, and the alignment is only conducted on the locations with the top two most *k*-mer hash references. Furthermore, although the original *GNUMAP* software supports multi-threaded computing within the same node using pthread, *GNUMAP-bs* is fully enabled to support MPI processing. This feature allows a large-scale alignment to be spread across multiple nodes in a cluster or supercomputing facility [28]. In this implementation, the genome is divided into equal parts across nodes, and then the same batches of reads are aligned by each node to their individual portion of the genome. Once the batch is completed, the nodes communicate via MPI to calculate the posterior probability scores. Because most of the CPU time is spent on the alignments, the communication overhead is relatively small, resulting in a highly efficient parallel algorithm.

## Competing interests

The authors declare that they have no competing interests.

## Authors’ contributions

QS, MJC, and WEJ conceived the study and developed the algorithm. CH and NLC implemented the algorithm and developed the software. CH and SC generated the simulated read dataset and analyzed both datasets. SSH, DTC, and BRC generated the human BS-seq datasets. CH and WEJ wrote the paper. All authors read and approved the final manuscript.
